# Case Report: Management of refractory granulomatous lobular mastitis with precision surgery and local irrigation

**DOI:** 10.3389/fsurg.2026.1851347

**Published:** 2026-09-09

**Authors:** Fuqing Ji, Mingkun Zhang, Haili Tang, Xulong Zhu, Lanhui Zhang, Teng Qi

**Affiliations:** 1 Department of Traditional Chinese Medicine, Xi’an No.3 Hospital, the Affiliated Hospital of Northwest University, Xi’an, Shaanxi, China; 2 Department of Thyroid, Breast, and Vascular Surgery, Xijing Hospital, The Fourth Military Medical University, Xi’an, Shaanxi, China; 3 Department of General Surgery, Tangdu Hospital, Air Force Medical University, Xi’an, Shaanxi, China; 4 Department of Surgical Oncology, Shaanxi Provincial People's Hospital, Xi'an, China; 5 School of Medicine, The Chinese University of Hong Kong, Shenzhen, Guangdong, China

**Keywords:** breast-conserving surgery, case report, dexamethasone, granulomatous lobular mastitis, precision surgery, refractory mastitis, therapeutic irrigation

## Abstract

**Background:**

Granulomatous lobular mastitis (GLM) is a rare chronic inflammatory breast disease with a high propensity for recurrence. Refractory cases that involve extensive abscess formation, sinus tracts, and failure of medical therapy remain difficult to manage.

**Case presentation:**

A 39-year-old multiparous woman presented with a left breast mass that had first been noticed approximately 6 months earlier and had progressively enlarged to involve multiple quadrants. The disease was complicated by abscess formation, areolar ulceration, and sinus tracts. Prior treatment with anti-inflammatory therapy, a course of oral corticosteroid, and a 4-month course of traditional Chinese medicine produced no durable improvement, and the mass subsequently ulcerated. Preoperative evaluation identified hyperprolactinemia and a Rathke cleft cyst on pituitary magnetic resonance imaging.

**Intervention and management:**

After preoperative prolactin control with bromocriptine, the patient underwent parenchyma-sparing surgery with thorough debridement of necrotic tissue across multiple quadrants. Mildly edematous glandular tissue and clinically involved ducts were preserved and opened to facilitate postoperative drainage. After glandular flap reconstruction, the surgical cavity was irrigated daily through two indwelling drains with 250 mL of normal saline containing dexamethasone acetate, initiated at 20 mg; the dose was reduced by 5 mg (one ampoule) every 3 days to a final dose of 5 mg, after which the cavity was rinsed with normal saline alone until the drains were removed. Syndrome-differentiated oral traditional Chinese medicine was given throughout the postoperative course.

**Results:**

The drainage fluid became clear and the drains were removed without residual purulent or necrotic material. Serial postoperative ultrasonography demonstrated progressive resolution of the inflammatory changes. At the 2-month follow-up, clinical examination showed no residual disease or recurrence, and the cosmetic outcome was satisfactory; the patient remained free of clinical recurrence during continued outpatient follow-up of approximately 2 years.

**Conclusion:**

Parenchyma-sparing surgery combined with local corticosteroid irrigation achieved short-term local control in a refractory case of GLM. The preliminary result warrants confirmation through longer follow-up and larger prospective studies.

## 1 Introduction

Granulomatous lobular mastitis (GLM), also known as idiopathic granulomatous mastitis, is a rare benign inflammatory breast disease that was first described as a lesion clinically simulating carcinoma (1). It typically affects parous women of reproductive age and is defined histologically by non-caseating granulomatous inflammation centered on the breast lobules (2, 3). The etiology remains incompletely understood, but an autoimmune process modulated by hormonal and immune factors, together with infection by Corynebacterium species, has been implicated (4–6). Because no internationally accepted treatment algorithm exists, management ranges from observation to corticosteroids, immunosuppressants, and surgery, and recurrence is common (7).

The clinical importance of GLM extends beyond its local morbidity. The disease frequently mimics breast cancer on clinical examination and imaging, which means that a confident histological diagnosis and the exclusion of malignancy and infection are prerequisites before any anti-inflammatory therapy is started (1, 2). Refractory GLM, defined by extensive abscess formation, multiple sinus tracts, and failure of conventional medical therapy, remains a particularly difficult scenario because radical excision risks breast deformity while medical therapy alone often fails to control the disease. We report a case of refractory GLM managed with a combined approach of parenchyma-sparing surgery and postoperative local corticosteroid irrigation. This report was prepared in accordance with the CARE guidelines for case reports (8, 9).

## 2 Case presentation

A 39-year-old multiparous woman (gravida 3, para 2) presented with a left breast mass that had been noticed approximately 6 months earlier. The mass was initially about 3 cm, firm, warm, and painful, and it progressively enlarged to involve the lower quadrant, the areola, and the upper quadrant. She had been evaluated at several hospitals; anti-inflammatory therapy and a course of oral methylprednisolone at another hospital produced no clear improvement, and a subsequent 4-month course of traditional Chinese medicine also failed to achieve remission, after which the mass ulcerated with abscess and sinus formation. Core needle biopsy at another hospital had confirmed granulomatous lobular mastitis.

**Figure 1:**
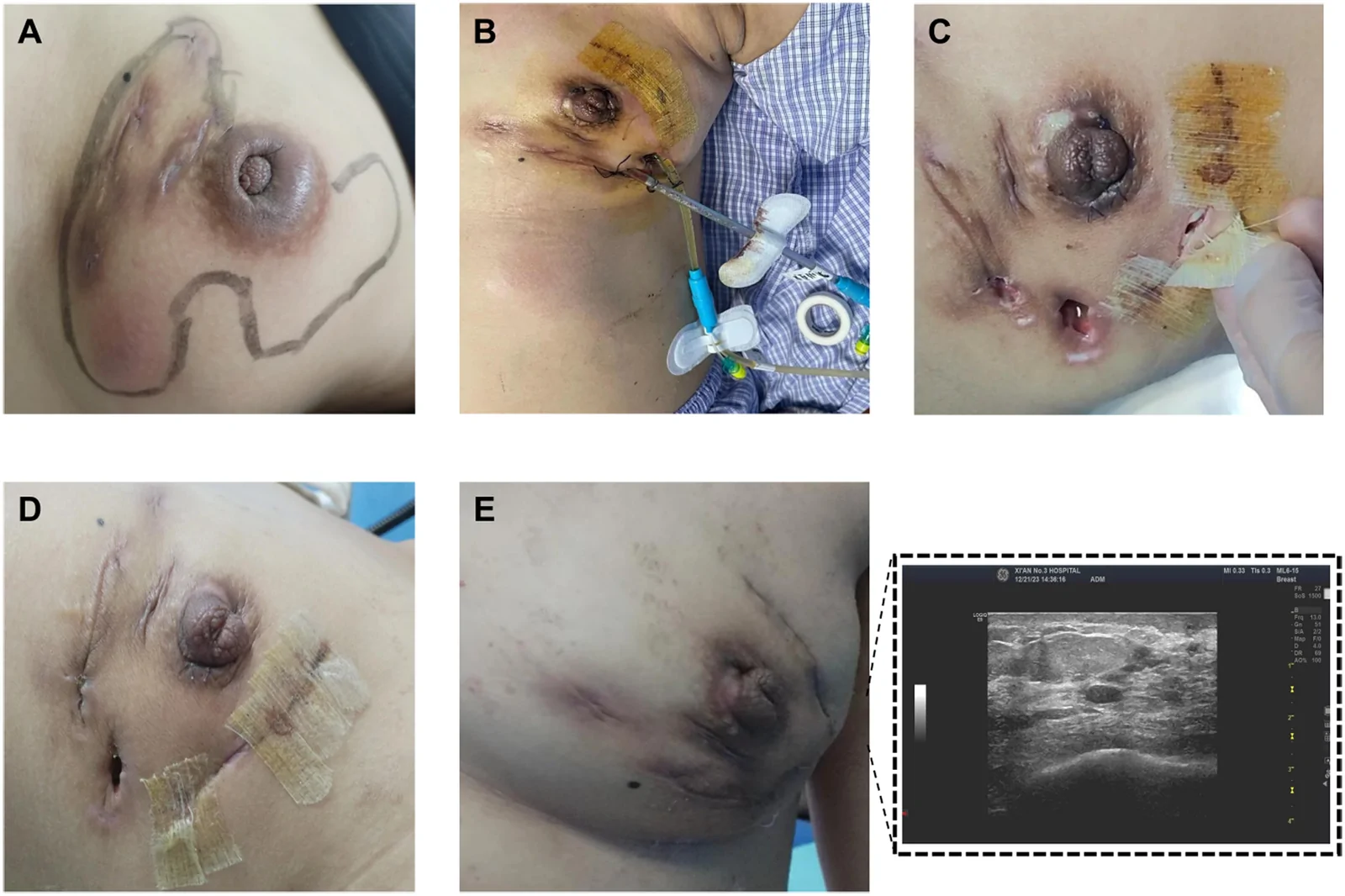
Clinical course and surgical management. **(A)** Preoperative appearance showing patchy erythema, ulceration, and sinus formation. **(B)** Early postoperative view with two irrigation drains in place. **(C)** Daily local irrigation with dexamethasone acetate through the drain using a tapering regimen. **(D)** Postoperative healing during the irrigation course. **(E)** Follow-up appearance with a satisfactory cosmetic result and no signs of recurrence, together with confirmatory ultrasonography showing resolution of the abscess (BI-RADS category 3).

Physical examination revealed inversion of the left nipple and an irregular, ill-defined area of induration measuring approximately 5 × 7 cm near the 12 o'clock position, with a 0.5 cm ulcerated opening at the 5 o'clock position. Localized erythema and peau d'orange changes were present, and enlarged lymph nodes were palpable in the left axilla (Figure 1A).

**Figure 2:**
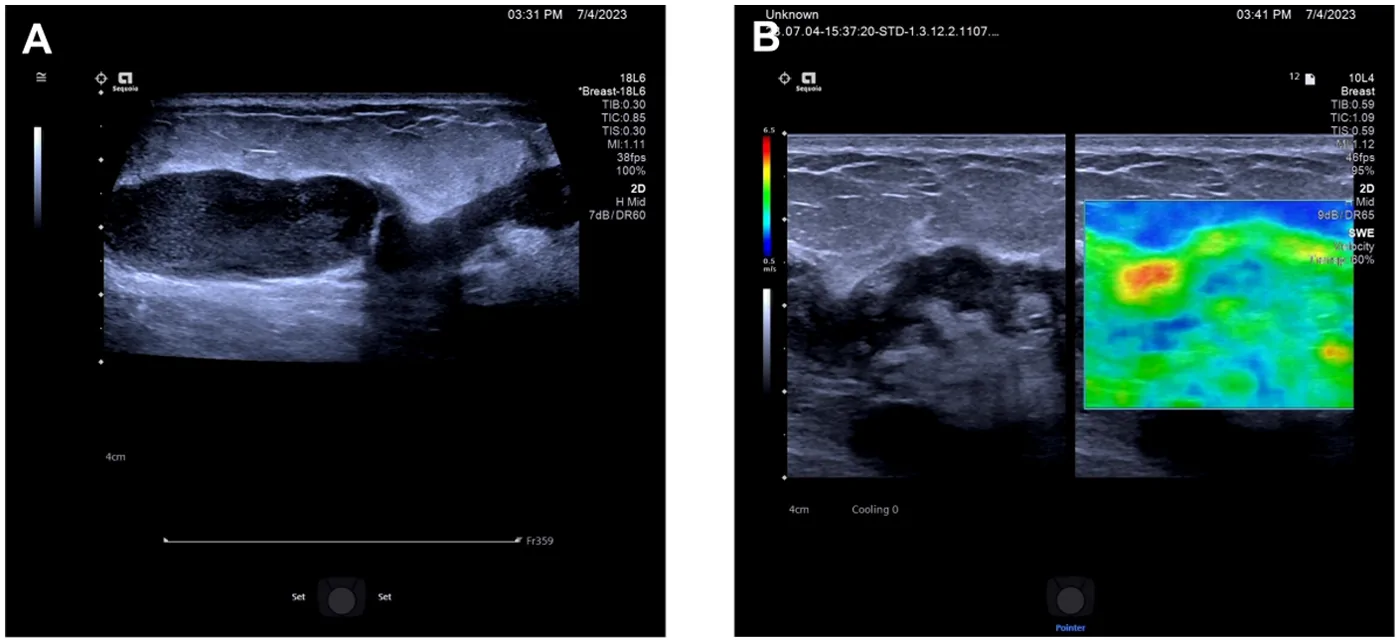
Preoperative ultrasonography of the breast. (A) Grayscale ultrasound showing a heterogeneous, predominantly hypoechoic lesion with irregular contours and internal echogenic material, suggestive of abscess formation in the clinical context. (B) Corresponding grayscale and elastographic images showing a heterogeneous elastographic pattern in the imaged region.

Breast ultrasonography demonstrated multiple heterogeneous hypoechoic areas embedded within a larger mixed-echo inflammatory field, the largest measuring 70.5 × 15.8 × 40.0 mm (7.05 × 1.58 × 4.0 cm) in the upper outer quadrant, with ill-defined borders and irregular morphology. Additional hypoechoic lesions were identified near the nipple (36.5 × 17.7 mm) and at the 3 o'clock position 2.5 cm from the nipple (23.7 × 12.6 mm), showing internal echogenic debris and vascular flow. Multiple sinus tracts and enlarged axillary lymph nodes were also noted, findings consistent with inflammatory change and abscess formation (Figure 2).

Laboratory evaluation showed a serum prolactin level of 21.41 ng/mL, and pituitary magnetic resonance imaging revealed a Rathke cleft cyst. A post-treatment increase in prolactin level is a recognized recurrence risk factor in GLM (10), and elevated prolactin was identified in a substantial proportion of patients in a large clinicopathologic series (11). Histopathological examination of the core biopsy confirmed chronic granulomatous inflammation with lymphocytic infiltration, histiocytic proliferation, and microabscess formation. A chronological summary of the episode of care is presented in Table 1 and Figure 1.

**Table 1:** Timeline of the episode of care.

Time Point	Event	Details
∼6 months before admission	Disease onset	Left breast mass, approximately 3 cm, firm, warm, and painful
∼5 months before admission	Outside-hospital pathology	Core needle biopsy: granulomatous lobular mastitis (GLM)
∼4–5 months before admission	Medical therapy	Anti-inflammatory therapy and oral methylprednisolone with no clear improvement; followed by oral traditional Chinese medicine (TCM) for 4 months, also without improvement; the mass ulcerated
∼2 weeks before admission	Outside-hospital ultrasonography	Mixed-echo inflammatory lesion (10.7 × 4.2 × 9.1 cm) in the left breast, representing the overall inflammatory field, BI-RADS category 3; reactive axillary lymphadenopathy
Day 0 (admission)	Admission and workup	Serum prolactin 21.41 ng/mL; pituitary MRI: Rathke cleft cyst; bromocriptine initiated; dietary modification; diagnostic biopsy confirmed GLM; infection and malignancy excluded
Day 2	Surgery	Parenchyma-sparing segmental resection with debridement of necrotic tissue, glandular flap reconstruction, and placement of two irrigation drains; intraoperative frozen section: inflammatory change
Days 3–10	Inpatient local therapy and TCM	Daily local dexamethasone acetate irrigation via drains; TCM consultation with oral herbal medicine, auricular acupressure, and acupoint application; discharged on oral TCM, tapering oral methylprednisolone, and calcium supplementation
Days 13–36	Outpatient irrigation and wound care	Continued irrigation and dressing changes; drains removed once drainage became clear; serial ultrasonography showed progressive resolution of inflammatory changes
Month 2	Clinical follow-up	No residual disease or recurrence; satisfactory cosmetic result
Month 5	Ultrasonography	Only small residual hypoechoic areas without abscess, BI-RADS category 3
Months 18–25	TCM outpatient follow-up	Syndrome-differentiated herbal therapy for postoperative constitutional regulation; no clinical evidence of recurrence at the last visit

## 3 Diagnostic assessment

**Figure 3:**
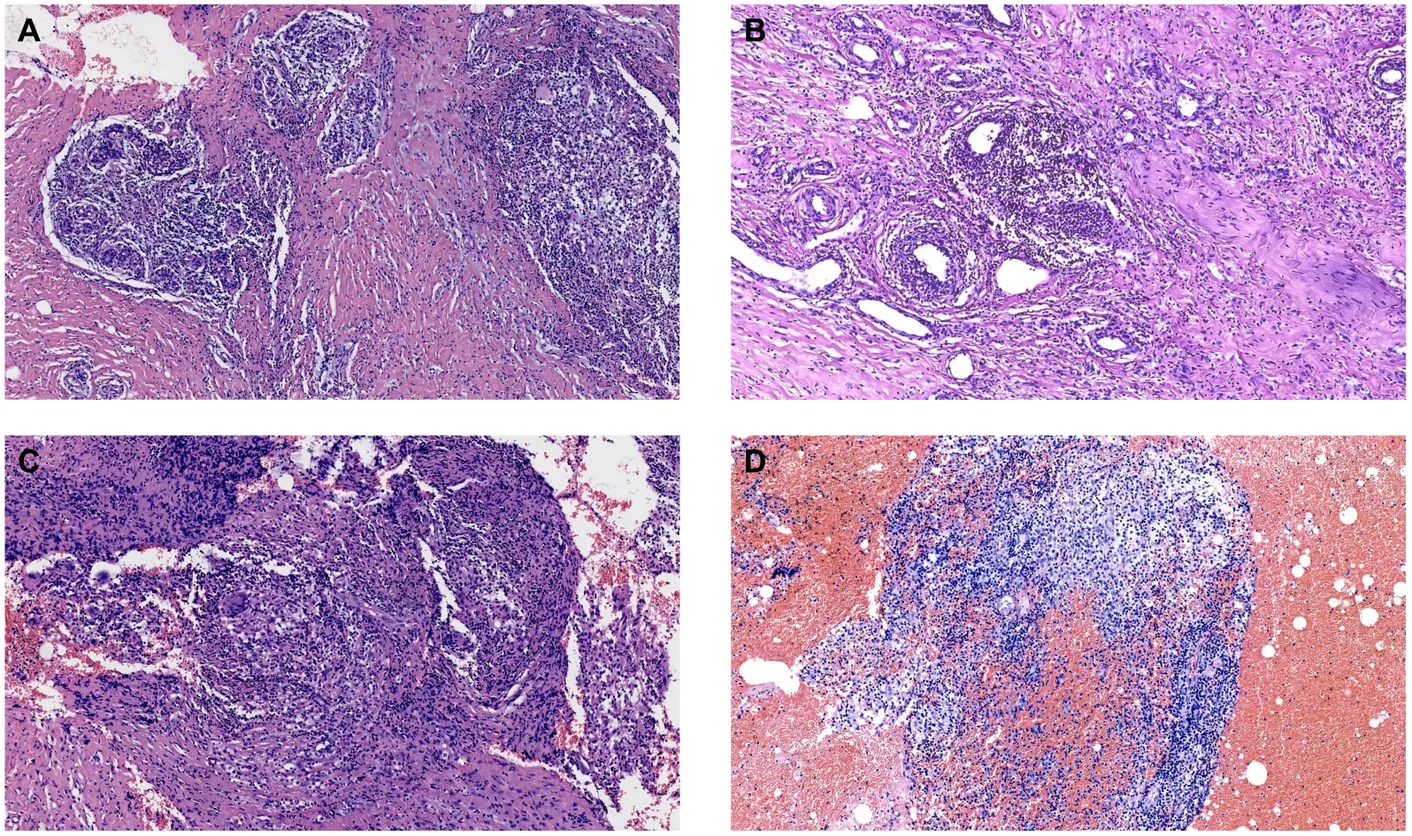
Representative histopathology of granulomatous lobular mastitis (hematoxylin and eosin). **(A)** Low-power view showing lobulocentric chronic granulomatous inflammation (original magnification ×100). **(B)** Periductal and perilobular lymphocytic and histiocytic infiltration with stromal fibrosis (×100). **(C)** Granuloma with multinucleated giant cells (×200). **(D)** Microabscess formation with neutrophils and histiocytes (×200).

The diagnosis of GLM rests on histopathology, and the differential diagnosis is broad. The most important considerations are malignancy, infection, and other granulomatous diseases. Because GLM can present as an irregular, fixed mass with nipple retraction, skin changes, and axillary lymphadenopathy, it is readily mistaken for breast cancer, and mastitis-like findings can occasionally represent the presentation of inflammatory breast cancer or ductal carcinoma *in situ* (12). Infection must likewise be excluded, because tuberculous mastitis and Corynebacterium-associated disease can produce a granulomatous picture that overlaps with GLM (6, 13). In this patient the diagnosis was established by core needle biopsy, which demonstrated the characteristic lobulocentric non-caseating granulomatous inflammation (Figure 3A), periductal and perilobular lymphocytic and histiocytic infiltration with stromal fibrosis (Figure 3B), granulomas with multinucleated giant cells (Figure 3C), and microabscess formation (Figure 3D), with no malignant cells identified. Staining and cultures were performed to exclude acid-fast bacilli, fungi, and Corynebacterium species, and tuberculosis screening was negative, consistent with current recommendations for the pathological diagnosis of GLM (14). The absence of caseating necrosis and the negative microbiological workup helped to exclude tuberculous mastitis before corticosteroid was delivered into the lesion. Although granulomatous mastitis can be associated with systemic features such as erythema nodosum, this patient did not show such findings (15).

## 4 Therapeutic intervention

Preoperative management included comprehensive evaluation, dietary modification, and bromocriptine to reduce the elevated prolactin level. Dietary modification was empirical and consisted of avoiding spicy and stimulating foods and other items that, in our clinical experience, have been associated with disease flares, although this practice is not supported by formal evidence. Surgical intervention combined a conventional incision with a spindle-shaped incision that included the ulcerated skin. Electrosurgical dissection was performed layer by layer with meticulous hemostasis. Necrotic tissue across multiple quadrants was thoroughly excised, whereas mildly edematous glandular tissue and clinically involved ducts were preserved and opened to facilitate postoperative irrigation. Intraoperative frozen section confirmed inflammatory change. Glandular flap reconstruction was performed after maximal preservation of breast parenchyma. The surgical cavity was irrigated with hydrogen peroxide, povidone-iodine, and normal saline, and two drains were placed for postoperative irrigation (Figure 1B). The incision was closed with absorbable sutures in a cosmetic layered fashion.

Postoperative management consisted of daily irrigation of the surgical cavity through the drains (Figure 1C). The irrigation solution consisted of 250 mL of normal saline containing dexamethasone acetate, initiated at 20 mg; the dose was reduced by 5 mg (one ampoule) every 3 days to a final dose of 5 mg (one ampoule), after which the cavity was rinsed with normal saline alone until the drainage became clear and the drains were removed. In addition, the patient received syndrome-differentiated oral traditional Chinese medicine together with auricular acupressure and acupoint application during hospitalization, and continued oral traditional Chinese medicine after discharge together with a tapering course of oral methylprednisolone and calcium supplementation. The herbal formula was modified according to syndrome differentiation in line with the Chinese expert consensus on the traditional Chinese medicine diagnosis and treatment of GLM; representative herbs used during follow-up included Astragalus membranaceus (Huangqi), Codonopsis pilosula (Dangshen), Angelica sinensis (Danggui), Agrimonia pilosa (Xianhecao), Bupleurum chinense (Chaihu), Citrus reticulata peel (Chenpi), Polygala tenuifolia (Yuanzhi), and Glycyrrhiza uralensis (Gancao) (16, 17). No procedure-related or drug-related adverse events were observed during the treatment period.

## 5 Follow-up and outcomes

The drainage fluid decreased progressively and became clear, and the drains were removed once no purulent or necrotic material remained. The wound healed without infection or dehiscence (Figure 1D). Serial postoperative ultrasonography showed progressive resolution of the residual inflammatory changes, and the examination performed approximately 5 months after surgery showed only small residual hypoechoic areas without abscess (BI-RADS category 3). At the 2-month follow-up, clinical examination showed no residual disease or recurrence, and the patient reported satisfaction with the cosmetic result (Figure 1E). The patient tolerated the irrigation well and adhered to the treatment schedule without interruption. She continued traditional Chinese medicine outpatient follow-up thereafter, and at the most recent visit approximately 2 years after surgery there was no clinical evidence of recurrence.

## 6 Discussion

This case illustrates a combined strategy for refractory GLM in which parenchyma-sparing surgery removed the necrotic burden while local corticosteroid irrigation targeted residual inflammation. The individual components have been described separately in the literature, but their combination is less well documented, and the rationale for each component deserves discussion.

Conservative management and observation are reasonable options for selected patients with mild GLM, because the disease can resolve spontaneously in a proportion of cases (18). For more extensive or refractory disease, systemic corticosteroids remain the most widely used first-line medical therapy, and methotrexate is reserved for patients who do not tolerate or respond to steroids (19). Our patient had already failed a course of oral corticosteroid before referral, which was one reason a different strategy was chosen. Surgical resection is an effective treatment for GLM (20), but it carries a risk of breast deformity when the excision is extensive, and complete excision is often impossible in a disease that is diffuse and multifocal (2). This tension between adequate disease clearance and cosmetic preservation is central to the management of GLM.

The use of locally delivered corticosteroid is supported by an emerging body of evidence. A systematic review and meta-analysis reported that local steroid administration is effective for idiopathic granulomatous mastitis (21), and topical steroid treatment has been shown to produce clinical and radiological improvement (22). Most relevant to our approach, a randomized controlled trial comparing systemic and intralesional steroid in recurrent or refractory granulomatous mastitis found that delivering corticosteroid directly to the lesion, rather than systemically, is a viable strategy for this population (23). Our decision to combine surgical debridement with local corticosteroid irrigation was based on this principle: the surgery removes the grossly necrotic tissue, while the locally applied corticosteroid suppresses the residual granulomatous inflammation that is thought to drive recurrence. In this case the irrigation was delivered through the drains as a tapered intracavitary protocol (20 mg of dexamethasone acetate in 250 mL of normal saline, reduced by 5 mg every 3 days to 5 mg, followed by normal saline rinsing), which extends the intralesional-steroid concept to the postoperative cavity. A minimally invasive approach combining indwelling catheter drainage with irrigation of the lesion has likewise been described for GLM, in which the cavity was irrigated with hydrogen peroxide, metronidazole, and dexamethasone through indwelling hoses (24). This reasoning is consistent with the concept that residual inflammatory foci left behind after surgery are an important source of relapse, and with the multidisciplinary, parenchyma-preserving philosophy that has been advocated for this disease (25).

The role of traditional Chinese medicine in GLM is increasingly recognized. A 2-year follow-up study reported favorable long-term outcomes with traditional Chinese medicine and analyzed recurrence and new occurrence rates together with risk factors (26). Chinese expert consensus documents provide guidance on syndrome differentiation and herbal treatment for GLM (16, 17). In our institution, we favor an integrated approach in which Western medicine provides rapid local control through surgery and corticosteroid, whereas traditional Chinese medicine is used to regulate the whole body and address the underlying constitutional imbalance; in this patient the postoperative herbal formula was adjusted to tonify qi and nourish blood according to her syndrome. This integrated approach was one reason we did not escalate to methotrexate, which is rarely used in our region and carries a burden of potential toxicity. It is important to acknowledge, however, that the superiority of an integrated approach over any single modality has not been established in comparative trials. A meta-analysis of treatments for GLM has described remission and recurrence outcomes across modalities, and a recent framework has proposed an endocrine-microbe-immunity model that may explain its pathogenesis and phenotype-oriented interpretation (27, 28).

Several limitations of this report must be emphasized. First, although the patient remained free of clinical recurrence during approximately 2 years of outpatient follow-up, the imaging confirmation of disease quiescence rests on the early postoperative period, and GLM has a protracted course with a tendency to late recurrence, so longer imaging follow-up remains desirable (7, 29, 30). A 5-year follow-up study and recurrence analyses have shown that GLM may relapse after apparently successful treatment, and the outcome of a subsequent pregnancy, if any, should also be monitored given the uncertain relationship between pregnancy and recurrence (31). Second, this is a single-case observation, and the findings cannot be generalized. The combination we describe is a preliminary experience rather than an established alternative to standard therapy.

## 7 Patient perspective

The patient had sought treatment at multiple hospitals over several months without durable improvement and was distressed by the recurrent abscesses and the progressive disfigurement of the breast. After the combined procedure she reported relief of symptoms, satisfaction with the breast appearance, and no significant pain during the irrigation. She also expressed a preference for a treatment that avoided the prolonged use of systemic immunosuppressants, which she had been concerned about during her earlier courses of care, and she valued the continued traditional Chinese medicine follow-up.

## 8 Conclusion

Parenchyma-sparing surgery combined with local corticosteroid irrigation achieved short-term local control with a satisfactory cosmetic result in a patient with refractory granulomatous lobular mastitis. The approach is biologically plausible and is supported by published experience with local corticosteroid therapy, but its long-term efficacy and its place relative to established treatments remain to be defined by longer follow-up and larger prospective studies.

## Data Availability

The original contributions presented in the study are included in the article/Supplementary Material, further inquiries can be directed to the corresponding authors.
